# Both Functional LTβ Receptor and TNF Receptor 2 Are Required for the Development of Experimental Cerebral Malaria

**DOI:** 10.1371/journal.pone.0002608

**Published:** 2008-07-09

**Authors:** Dieudonnée Togbe, Paulo Loureiro de Sousa, Mathilde Fauconnier, Victorine Boissay, Lizette Fick, Stefanie Scheu, Klaus Pfeffer, Robert Menard, Georges E. Grau, Bich-Thuy Doan, Jean Claude Beloeil, Laurent Renia, Anna M. Hansen, Helen J. Ball, Nicholas H. Hunt, Bernhard Ryffel, Valerie F. J. Quesniaux

**Affiliations:** 1 University of Orléans and CNRS, Molecular Immunology and Embryology UMR6218, Orleans, France; 2 CNRS CBM (Centre de Biophysique Moléculaire), Orleans, France; 3 Institute of Infectious Disease and Molecular Medicine, Cape Town, South Africa; 4 University of Duesseldorf, Duesseldorf, Germany; 5 Pasteur Institute Paris, Paris, France; 6 The University of Sydney, Department of Pathology, Camperdown, Australia; 7 Institut Cochin, Université Paris Descartes, CNRS (UMR 8104), Paris, France; 8 Inserm, U567, Paris, France; Queensland Institute of Medical Research, Australia

## Abstract

**Background:**

TNF-related lymphotoxin α (LTα) is essential for the development of *Plasmodium berghei* ANKA (PbA)-induced experimental cerebral malaria (ECM). The pathway involved has been attributed to TNFR2. Here we show a second arm of LTα-signaling essential for ECM development through LTβ-R, receptor of LTα1β2 heterotrimer.

**Methodology/Principal Findings:**

LTβR deficient mice did not develop the neurological signs seen in PbA induced ECM but died at three weeks with high parasitaemia and severe anemia like LTαβ deficient mice. Resistance of LTαβ or LTβR deficient mice correlated with unaltered cerebral microcirculation and absence of ischemia, as documented by magnetic resonance imaging and angiography, associated with lack of microvascular obstruction, while wild-type mice developed distinct microvascular pathology. Recruitment and activation of perforin^+^ CD8^+^ T cells, and their ICAM-1 expression were clearly attenuated in the brain of resistant mice. An essential contribution of LIGHT, another LTβR ligand, could be excluded, as LIGHT deficient mice rapidly succumbed to ECM.

**Conclusions/Significance:**

LTβR expressed on radioresistant resident stromal, probably endothelial cells, rather than hematopoietic cells, are essential for the development of ECM, as assessed by hematopoietic reconstitution experiment. Therefore, the data suggest that both functional LTβR and TNFR2 signaling are required and non-redundant for the development of microvascular pathology resulting in fatal ECM.

## Introduction

Cerebral malaria is a frequent cause of death in children and young adults infected with Plasmodium falciparum [1], which is characterized by the sequestration of parasitized erythrocytes in cerebral blood vessels [2], [3]. TNF has been originally implicated in pathogenesis of the neurological complication [4]. In patients with cerebral malaria disease severity has been correlated with TNF serum levels [5]–[7]. In experimental murine malaria the administration of TNF neutralizing antibodies prevented experimental cerebral malaria (ECM) [8]. Mice with a combined inactivation of TNF and LTα were to be resistant to ECM development [9]. Recent data suggest that LTα rather than TNF is responsible for the development of microvascular damage resulting in the neurological malaria complication [10].

Lymphotoxin alpha is a member of TNF superfamily and the closest TNF relative. It exists as a soluble homotrimer (LTα3) or forms membrane-bound heterotrimers with the membrane associated lymphotoxin beta (LTβ) [11], [12]. Both homotrimeric TNF and LTα3 interact with two receptors, TNFR1 (CD120α) and TNFR2 (CD120β), whereas membrane bound LTαβ heterotrimers function through the engagement of LTβR [13], [14]. LTβR binds not only LTαβ heterotrimers but also LIGHT homotrimers, another TNF family member. Both LIGHT and LTα3 bind herpes virus entry mediator (HVEM), a fourth member of TNFR superfamily. The role of soluble LTα3, of LTαβ and LIGHT in pathogenesis is not fully understood.

Mice with complete inactivation of TNF or TNFR1 gene are unable to mount a protective response against various pathogens [15], but develop ECM, while TNFR2 deficient mice are resistant to ECM development [16]. In order to assess the role of membrane LTαβ heterotrimer signaling we studied ECM development in LTβR deficient mice [17] and further extended the investigation to LIGHT, using LIGHT gene deficient mice [18].

Here we report that absence LTβR prevented ECM development. Magnetic resonance imaging (MRI) and angiography (MRA) were used to verify the lack of ischemia and microvascular pathology in these mice. Therefore, the data suggest that both functional LTβR and TNFR2 axis are required for the development of microvascular pathology resulting in fatal ECM. The disruption of either axis prevents cerebral microvascular disease.

## Results

### Disruption of LTβR signaling protects from fatal ECM development

LTα and TNFR2 signaling have been shown to be critical for Plasmodium berghei ANKA (PbA) induced ECM, as mice deficient for either LTα or TNFR2 are resistant to cerebral malaria, while TNFR1 is dispensable [10], [16]. Since LTα exists either as soluble homotrimer or heterotrimer in association with membrane bound LTβ, we asked whether signaling of membrane LTαβ through LTβR may be involved in ECM development.

**Figure 1:**
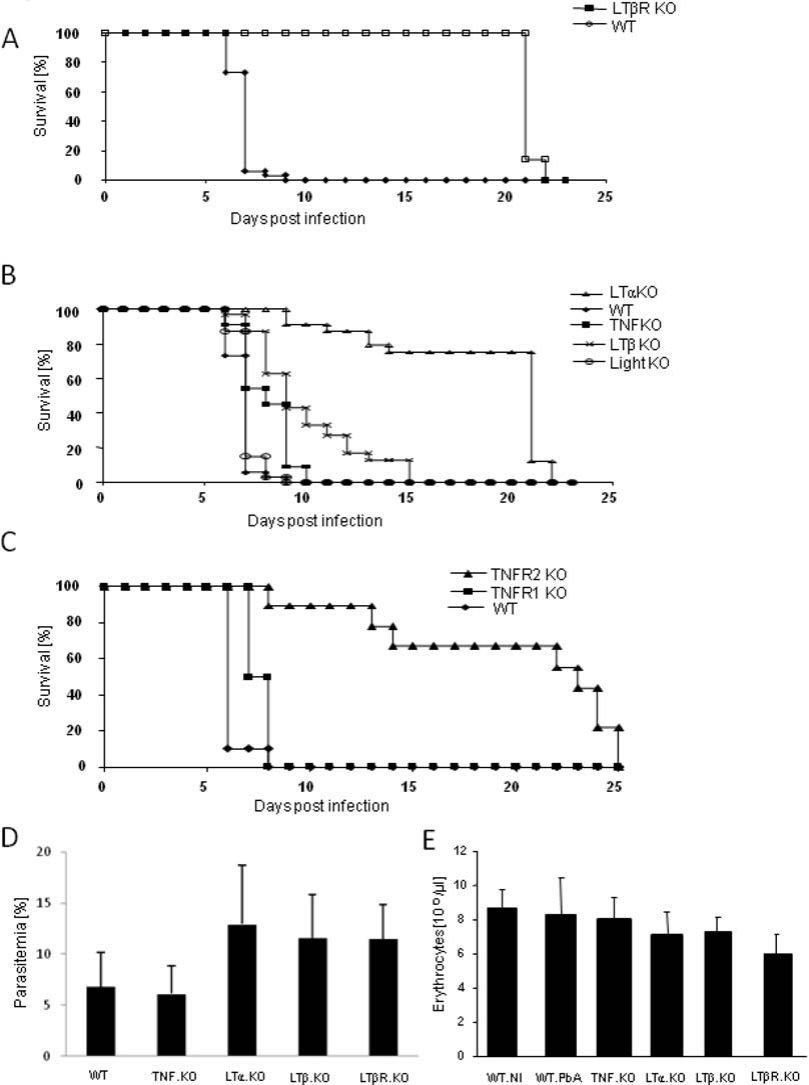
LTβR deficient mice do not develop PbA induced ECM. C57BL/6 (WT) and mice deficient for LTβR (A), LTα, LTβ, LIGHT or TNF (B) , TNFR1 or TNFR2 (C) were infected with 10^6^ *P. berghei ANKA* parasitized red blood cells and their survival monitored daily. At day 7, parasitemia (D) was significantly increased in LTα, LTβ and LTβR-deficient mice (p<0.01) and red blood cell counts (E) were significantly reduced in LTbR-deficient mice (p<0.0001) and less so in LTα or LTβ deficient mice (p<0.01). Results are from n = 14–33 mice per genotype pooled from five independent experiments. Logrank test analysis of the Kaplan-Meyer curves indicated p values <0.0001 for each genotype compared to WT, with the exception of LIGHT-deficient mice which was non significant (0.3) and TNF-deficient mice (<0.05).

We show here that LTβR deficient mice did not develop ECM and survived more than 20 days, while C57BL/6J wild type mice developed typical neurological signs of CM, including postural disorders, ataxia, impaired reflexes and loss of grip strength, progressive paralysis and coma, and succumbed within a week after infection with 5–10% of parasitized erythrocytes (*Figure 1A*). In order to compare directly the requirement for different members of the TNF family, mice deficient for TNF, LTα, LTβ, LIGHT, TNFR1 or TNFR2 were infected with blood stage PbA. LTα and TNFR2 deficient mice resisted to ECM development (*Figure 1B, C*), with no neurological signs up to day 22–25, at which time they succumbed of high parasitaemia and anemia (*Figure 1D,E* and data not shown), consistent with published data [16]. In contrast, mice deficient for TNF, LIGHT or TNFR1 developed neurological signs and succumbed to ECM within 7–10 days (*Figure 1B, C*). LTβ deficient mice were more susceptible than LTα deficient mice, although they did not show neurological signs in the first week of infection, and developed slightly higher parasitemia and anemia than wild-type mice (*Figure 1D, E*). Therefore, our data demonstrate that LTβR signaling is necessary for ECM development.

### Absence of ECM signs in LTβ-R deficient mice correlates with MRI and MRA based morphological analysis

Magnetic resonance imaging (MRI) is a non-invasive tool to investigate neurological disease processes in humans. In the murine experimental cerebral malaria model using MRI allow a semi-quantitative analysis of brain swelling, microvascular pathology due to obstruction of small vessels with parasitized erythrocytes and leukocytes endothelial damage, edema and focal ischemia [10], [16], [19]. Mice deficient for LTβR, LTαβ–deficient mice used as ECM resistant controls, and wild-type C57BL/6 or TNF-deficient mice used as ECM sensitive controls, were examined by MRI at day 7, when wild-type mice are dying with acute ECM, while LTβR and LTαβ–deficient mice were all alive without neurological signs.

**Figure 2:**
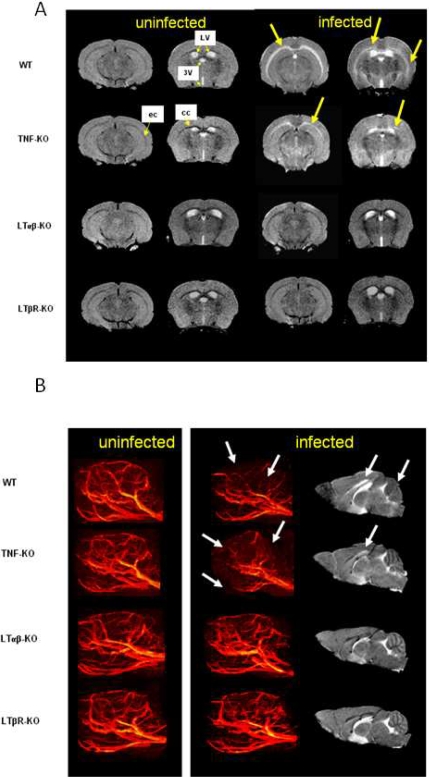
MRI analysis of PbA induced morphological changes. A. MRI of uninfected and infected mice, 7 days post infection with 10^6^ *P. berghei ANKA* parasitized red blood cells: Distinct anomalies are visible in C57BL/6 (WT) and TNF mice, namely edema formation, apparent as a bilateral hyperintense signal as indicated by arrows in the right 2 sets of photographs at the corpus callosum (c.c.) and external capsule (e.c.), while LTαβ and LTβR deficient mice do not show these alterations. LV and 3V indicate the lateral and third ventricles, respectively. Images correspond to the stereotaxic position Bregma −2.92 and −0.58 mm. B. Magnetic Resonance Angiography: when compared to uninfected mice (left panel) vascular blood flow perturbations (shown by arrows) are evident in WT and TNF-KO mice, 7 days post infection with 10^6^ *P. berghei ANKA* parasitized red blood cells, while LTαβ and LTβR deficient mice seem unaffected (middle panel). Sagittal MR images obtained from the same infected mice (right panel) indicate the main MRI alterations: oedema formation and cerebellar damage (shown by arrows). Brain swelling has been observed only in C57BL/6 and TNF-KO mice. MRA 3D reconstructions are shown in supplementary data.

Typical MRI and MRA images of uninfected and infected mice are shown in *Figure 2A and 2B*, respectively. While wild-type C57BL/6 and TNF-deficient mice presented signs of ischemic brain damage and vascular blood flow perturbations upon blood stage PbA infection, LTαβ and LTβR deficient mice displayed a normal MRI. Overall, mice deficient for LTaβ (*n* = 6) or LTβR (*n* = 7) showed unaltered MRI and MRA signals. By contrast, wild-type mice showed altered MRI, observable as a bilateral hyperintense signal at the corpus callosum and external capsule (*Figure 2A*), and a remarkable vascular blood flow perturbations was detected in wild-type mice (*Figure 2B*). In addition, brain swelling was observed in infected wild-type C57BL/6 mice during acute cerebral malaria. TNF deficient mice showed an intermediate phenotype with an altered MRI and MRA perturbation in infected mice analyzed. Non-infected wild-type or gene deficient mice presented normal MRI and MRA phenotype (*Figure 2A, B*). MRA 3D reconstitutions available in supplementary data show the clear difference in brain blood flow between non infected wild-type mice (*Video S1_B6_NI*), 7 day PbA infected wild-type (*Video S2_B6_PbA*) and TNF-deficient mice (*Video S3_TNF ko_PbA*) on the one hand, and mice deficient for LTβR (*Video S4_LTαβ ko_PbA*) or LTαβ (*Video S5_LTβR ko_PbA*), on the other hand.

Therefore, LTαβ-LTβR signaling is necessary to develop ischemic brain damage and vascular blood flow perturbations.

### LTβR deficient mice do not develop microvascular lesions

**Figure 3:**
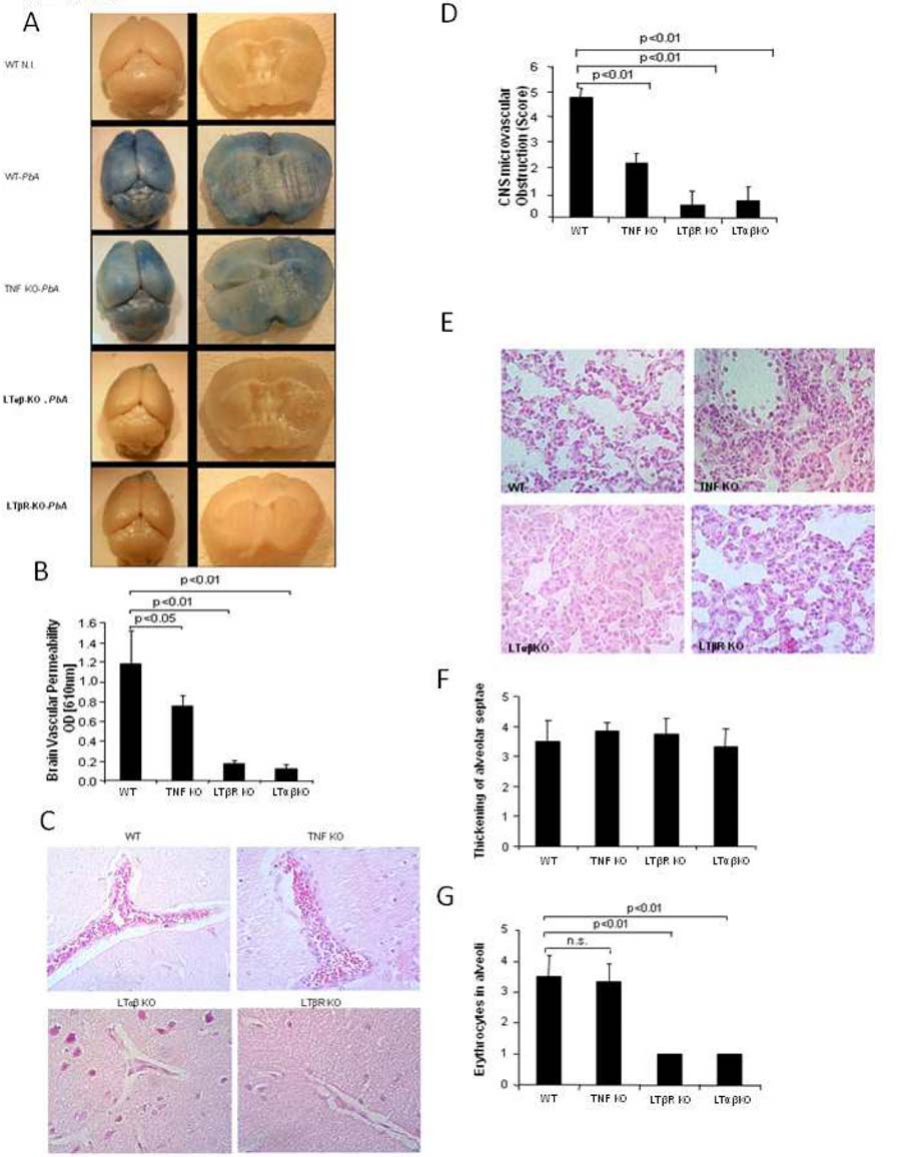
LTβR deficient do not develop ECM-associated brain vascular leakage. Vascular leak as assessed by the blue discoloration of the brain of wild-type control C57BL/6 (WT), TNF, LTα or LTβR deficient mice injected intravenously with 1% Evans blue on day 7 with severe ECM (A). Mice were infected with 10^6^ *P. berghei ANKA* parasitized red blood cells as in Fig. 1. Quantitative assessment of brain capillary permeability using Evans blue extravasation in formamide was measured by Absorbance at 610 nm in brain extract (B). Microvascular damage with mononuclear cell adhesion and perivascular hemorrhage in the brain of WT and TNF deficient mice, but not LTα, nor LTβR deficient, micrograph 400×, H&E stained brain sections (C). Semi-quantitative score of brain infiltration and hemorrhages (D). Microvascular damage of lung alveolar septae of B6 and TNF deficient mice, but not of LTβR deficient, micrograph 400×, H&E stained brain sections (E). semi-quantitative score of lung capillary (F–G). The data morphological data are from one representative out of 4–5 mice per group, and calculated date represent the mean±SD of n = 4 mice per group (n.s., not significant).

In order to validate functionally the data obtained by MRI, we investigated microvascular lesions in the brain. Microvessel obstruction and disruption, which leads to increased protein extravasation from the capillary bed, was quantified by Evans blue leakage into the tissue. PbA infected wild-type or TNF deficient mice showed a strong vascular leak of Evans blue, while mice deficient for LTαβ or LTβR had no discoloration of the brain parenchyma (*Figure 3A*), with limited Evans blue vascular leak (*Figure 3B*), suggestive of an intact blood-brain barrier.

Microscopically, the vascular leak is due to microvascular lesions in the brain with perivascular hemorrhage and intravascular accumulation of mononuclear cells and infected erythrocytes (*Figure 3C*), which was completely absent in LTαβ and LTβR deficient mice. The severity of microvascular obstruction and local hemorrhage in the brain were assessed semi-quantitatively, and clearly demonstrate significant differences. The information obtained by MRI was compared to that derived from the microscopic examination of the brain. There was a good agreement of the predicted brain pathology by MRI and microscopic investigation.

Since microvascular disease occurring in PbA infected mice presenting with CM is not limited to the brain, we also investigated the pulmonary microvascular beds. While the lungs of wild-type and TNF-deficient mice displayed congested septal capillaries, hemorrhages and interstitial edema, LTαβ or LTβR deficient mice had no evidence of microvascular pathology, with less erythrocytes in the alveoli (*Figure. 3E, G*). However, wild-type, TNF, LTαβ and LTβR deficient mice had comparable thickening of alveolar septae (*Figure 3F*).

Therefore, the data suggest that disruption of LTαβ – LTβR signaling prevents microvascular pathology induced by PbA blood stage infection.

### Reduced sequestration of effector T lymphocytes in the brain of PbA infected LTβR deficient mice

**Figure 4:**
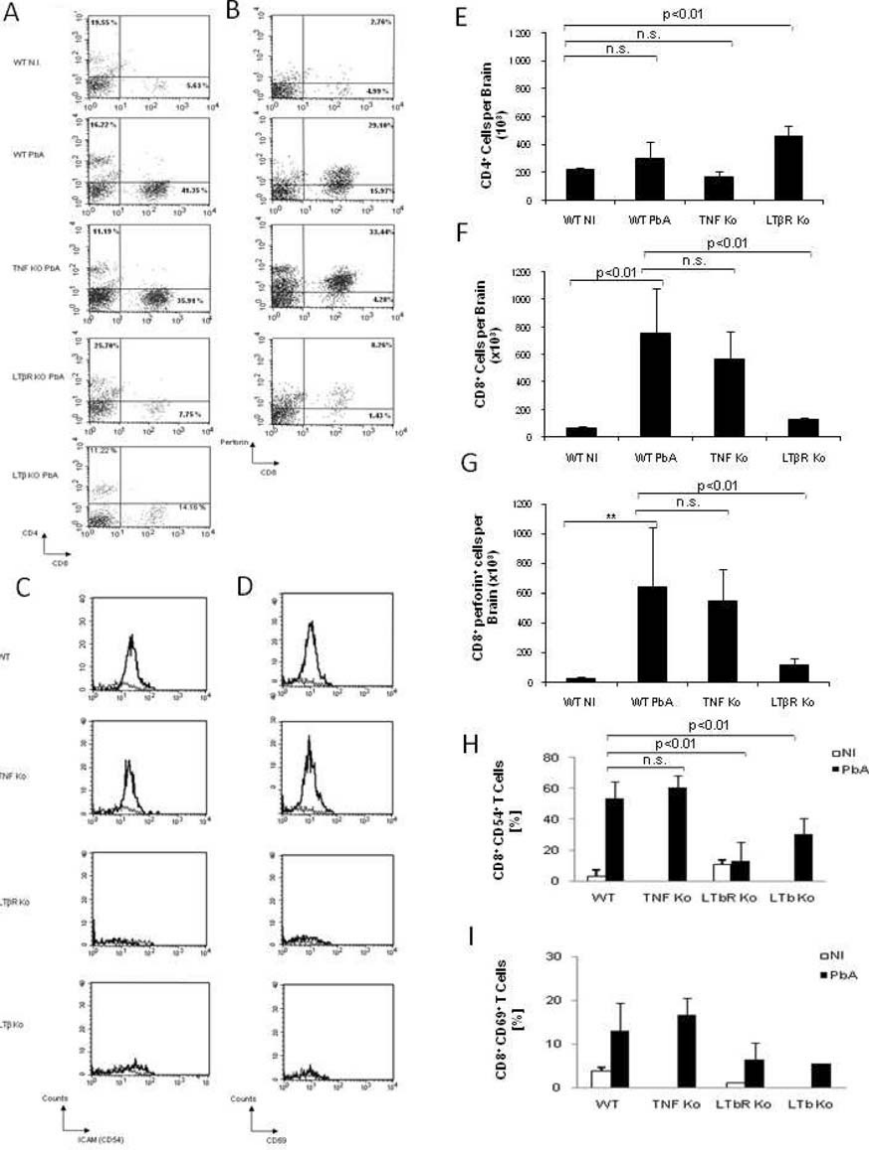
Reduced CD8^+^T lymphocytes infiltration in the brain of PbA-infected LTβR deficient mice. Flow cytometric analyses of brain-sequestered leukocytes from uninfected wild-type mice (WT NI), PbA-infected wild-type mice (WT PbA) and TNF deficient mice with severe ECM, and infected, ECM resistant LTβ and LTβR deficient mice tested on the same day. Mice were infected with 10^6^ *P. berghei ANKA* parasitized red blood cells as in Fig. 1. Representative dot blots of CD4 and CD8 staining (A) CD8 and perforin double staining (B). Activation markers CD54 (C) and CD69 (D) on CD8^+^ cells are shown for non infected (thin line) and PbA infected (thick line) WT, TNF-, LTβR- and LTβ–deficient mice, as indicated. Bargraphs of CD4^+^ (E), CD8^+^ (F), CD8^+^ Perforin^+^ (G), CD8^+^ CD54^+^ (H), and CD8^+^ CD69^+^ (I) lymphocytes represent the mean±SD of n = 3–4 pools of 3–4 mice per group, and are from one representative of two independent experiments (n.s., not significant).

Trafficking and recruitment of effector T lymphocytes into the brain are clearly necessary for the development of the pathology associated with ECM [20], [21]. We next characterized brain-sequestered leukocytes, in wild-type and TNF deficient mice with severe neurological symptoms after PbA infection and in LTβR or LTβ deficient mice at the same day after infection. Similar numbers of CD4^+^ T cells were recovered from uninfected and infected wild-type mice, as well as from PbA infected TNF or LTβ deficient mice (*Figure 4A, E*). Interestingly, the strong increase in brain CD8^+^ T cells seen in wild type or TNF deficient mice that develop cerebral malaria was essentially absent in PbA-infected LTβR or LTβ deficient mice (*Figure 4A, F*). We then analysed the functional state and cytotoxic potential of brain-sequestered CD8^+^ T cells from PbA-infected mice (*Figure 4B, G*). Most brain CD8^+^ T cells expressed perforin in PbA-infected wild-type or TNF deficient mice, but ECM resistant LTβR deficient mice sequestered far less perforin^+^ CD8 T lymphocytes (*Figure 4B, G*). Moreover, we show that LTβR or LTβ deficient mice have lower numbers of ICAM/CD54^+^ and CD69^+^ CD8 T cells than wild type or TNF deficient mice upon PbA infection (*Figure 4C,D, H, I*). LTβ deficient mice were only partially protected and succumbed to lethal ECM a few days later than the wild-type mice.

In conclusion, these data suggest a pathogenic role of activated effector CD8^+^ T lymphocytes recruited in brain of wild-type and TNF deficient mice developing ECM, whereas the ECM resistance in LTβR deficient mice is associated with strongly reduced sequestration of CD8^+^ T lymphocytes.

### Parasitaemia is LTαβ-LTβR signaling independent

**Figure 5:**
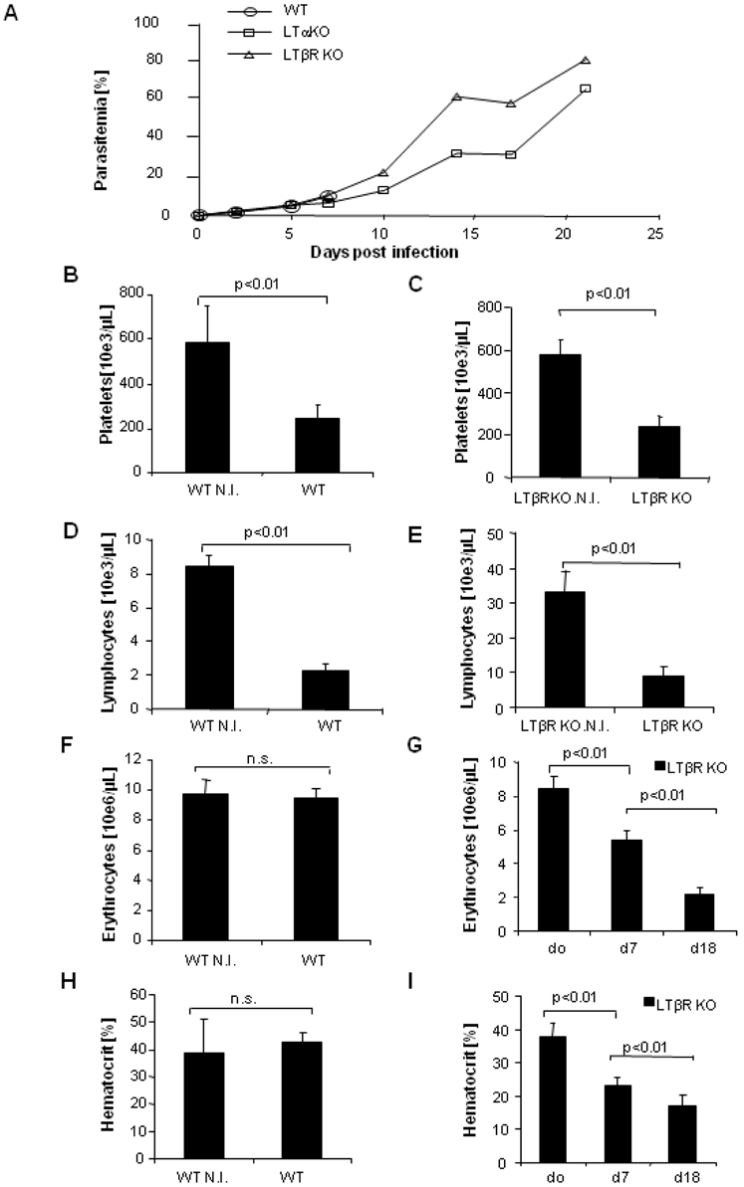
Parasitaemia and hematological alterations LTβR *deficient mice*. The percentage of parasitized erythrocytes in the peripheral blood was determined up to day 22 for ECM resistant mice (A) after infection with 10^6^ red blood cells parasitized by EGFP expressing *P. berghei ANKA*, by flow cytometry analysis of GFP fluorescent erythrocytes. Total platelet, lymphocyte and erythrocyte counts and hematocrit were determined in the peripheral blood of non infected (N.I.) and 7 day infected C57BL/6 and LTβR deficient mice (B–I). Anemia develops in LTβR deficient mice on day 18 after infection (I). The data represent the mean±SD of n = 5–12 mice per group from two independent experiments (n.s.; not significant).

We then asked whether parasitaemia and anemia are influenced in the absence of LTβR signaling. Parasitaemia was analyzed by flow cytometry using GFP transfected parasites [22]. Parasitaemia is usually low (less than 10%) at the time of ECM onset. Absence of LTβR had no effect on parasitaemia as compared to wild-type mice at day 7 (*Figure 5A*). However, mice deficient for either LTα or LTβR developed extremely high parasitaemia of about 70% by day 20 (*Figure 5B*), consistent with published data in LTα deficient mice [10].

The hematological investigations at 7 days after infection revealed distinct reductions of platelet counts in C57BL/6 mice as compared to non-infected mice (*Figure 5C*), as reported [23], and we show a similar thrombocytopenia in the ECM resistant LTβR deficient mice (*Figure 5D*). The latter finding suggests that thrombocytopenia in LTβR deficient mice is not an indicator of platelet sequestration in microvessels in this model.

The total white blood cell counts decreased after 7 days of PbA infection in wild type mice, and this was largely attributed to a decrease in lymphocytes (*Figure 5E*), while monocyte and neutrophil counts were essentially unaffected (data not shown). LTβR deficient mice also experienced a marked decrease in lymphocyte counts (*Figure 5F*).

Finally, no significant anemia developed in wild-type mice 7 days post-infection, the erythrocyte count was still in the normal range (*Figure 5G*) as well as the hematocrit (*Figure 5I*) and hemoglobin concentrations (data not shown). Erythrocyte counts and hematocrit were slightly decreased on day 7 in LTβR deficient mice (*Figure 5H, J*). The ECM resistant, LTβR deficient mice further developed dramatic anemia (*Figure 5H*), similar to LTα deficient mice (1.34±0.30×10^6^ RBC/µL), and probably succumb of general hypoxia after 3 weeks.

In conclusion, the resistance to ECM development of LTα and LTβR deficient mice was not attributable to decreased parasitaemia or thrombocytopenia, and the ECM resistant mice developed very high parasitaemia and died of severe anemia within 3 weeks.

### Absence of LTβR expression on stromal cells determines resistance to ECM

**Figure 6:**
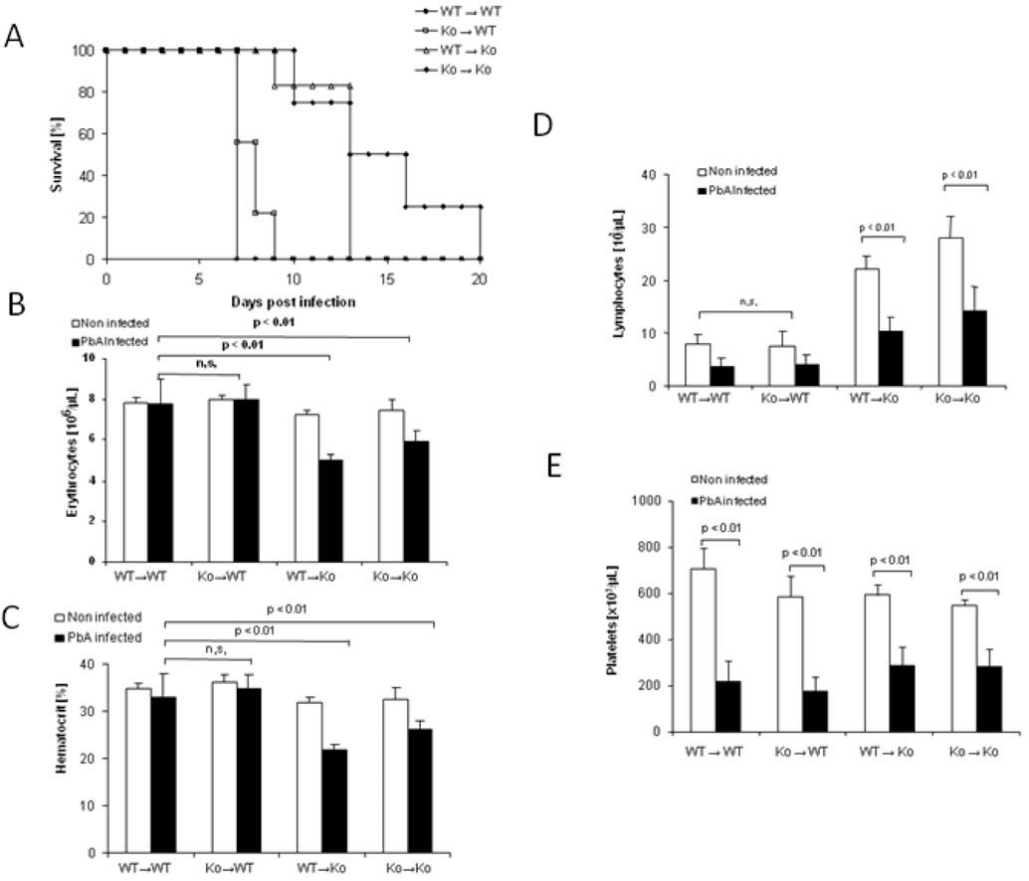
LTβR expressed on radioresistant stromal cells is critical for the development of ECM. C56BL/6 (WT) or LTβR deficient (KO) recipient mice were lethally irradiated and reconstituted with bone marrow cells from WT or KO mice as indicated (donor BM→recipient mice). The resulting WT→WT, WT→KO, KO→WT and KO→KO chimeric mice were infected with 10^6^ parasitized erythrocytes and survival was recorded (A). Hematological parameters such as erythrocyte counts (B), hematocrit (C), lymphocyte (D) and platelet (E) counts were determined on day 7 in naive and *P berghei Anka* (PbA) infected mice. The data represent the mean±SD of n = 5 mice per group (n.s., not significant).

To assess the cellular source of LTβR that mediates resistance toward ECM development, we generated bone marrow chimeric mice. Wild-type C57BL/6 and LTβR deficient mice were lethally irradiated and engrafted with bone marrow from either C57BL/6 mice or LTβR deficient mice [24], [25]. After infection with 10^6^ *P. berghei ANKA* parasitized erythrocytes, wild-type mice reconstituted with LTβR deficient bone marrow died as rapidly as wild-type mice reconstituted with wild-type bone marrow (*Figure 6A*). In contrast, LTβR deficient mice reconstituted with wild-type bone marrow displayed protection to ECM comparable to LTβR deficient mice reconstituted with LTβR deficient bone marrow, and further developed severe fatal anaemia, but no cerebral syndrome. Erythrocyte counts and hematocrit were already decreased on day 7 in LTβR deficient mice reconstituted with either LTβR deficient or wild-type bone marrow (*Figure 6B,C*), similar to non reconstituted LTβR deficient mice (*Figure 5H, J*). Platelet and lymphocyte count decrease seen 7 days after infection were largely dependent on the recipient mice (*Figure 6D, E*), and were similar to non-reconstituted mice (*Figure 5C–F*). Since the resistance of LTβR deficient mice to ECM development was not corrected by wild-type bone marrow reconstitution, the data suggest that LTβR expression on radioresistant, stromal probably endothelial cells determines the sensitivity to ECM and bone marrow cells from LTβR can not transfer ECM resistance to sensitive wild-type mice.

## Discussion

The present data demonstrate that interruption of LTβR signaling abrogates the microvascular disease leading to fatal ECM, which sheds a new light on the pathogenesis of cerebral malaria. Indeed, since LTα and TNFR2 signaling are also required for ECM development, we propose that both functional TNFR2 and LTβR axes are required for the development of microvascular pathology resulting in fatal ECM. The disruption of either axis prevents cerebral microvascular alterations, indicating that the two signaling pathways are non-redundant.

Signaling through TNF receptors activate the classic NF-κB/RelA pathway mediated by the inhibitors IκB-α, -β and -ε, leading to the expression of a large number of genes involved in inflammatory responses, cell adhesion or cell proliferation. In addition to the classic NF-κB/RelA pathway, LTβR signaling activates NF-κB through an alternative, non-canonical NIK-IKK1-dependent route that results in the activation of RelB/NF-κB2. The alternative pathway NF-κB is activated by a limited set of receptors including LTβR, CD27, CD40, BAFFR, RANK, but not by TNF receptors. A recent study identified p100 as a fourth IκB protein that is required and sufficient for non-canonical NF-κB signaling downstream of NIK and IKK1, while it acts also as a signal transducer of NF-κB/RelA [26]. P100 was proposed as an integrator, involved in a cross-talk between canonical and non-canonical NF-κB inducing signaling that result in qualitative changes in the expression of inflammatory genes [26]. Examples of dual requirement for TNFR and LTβR stimulation were described for the in vitro generation of fibroblast reticular cells from lymph node tissue [27].

Although LTα deficient mice are resistant to ECM, LTβ deficient mice were slightly less protected and absence of LIGHT did not prevent ECM. A similar apparent discrepancy, with milder phenotype for either LTβ or LIGHT deficient mice as compared to LTα or LTβR deficient mice, was reported previously for lymphoid organogenesis [18]. Evidence for a cooperative role for LIGHT and LTβ, existence of a weak LTα3-LTβR interaction or of an additional ligand for LTβR were proposed [18]. Here, LIGHT, another LTβR ligand appears to be redundant in pathogenesis of ECM to blood stage PbA infection.

We investigated several pathophysiological aspects of CM in LTβR-deficient mice and show that the absence of clinical signs of ECM is associated with normal cerebral perfusion as assessed by MRI. The in vivo imaging data correlated with absence of microvascular disease with accumulation of mononuclear cells, vascular leak, hemorrhage and inflammation in the brain. Further, platelet adherence to cerebrovascular endothelium was absent in LTβR-deficient mice (data not shown), which is an important factor in ECM development as reported before [28]. Further pulmonary microvascular damage with alveolitis, plasma and erythrocyte leak in the alveolar space seen in control mice was also reduced in LTβR-deficient mice. Therefore, PbA induced microvascular damage in both lungs and brain is LTβR signaling dependent.

A previous report demonstrated a sex dependence of LTβR deficient mice infected with *Plasmodium chabaudi* [29], therefore we investigated whether males and females LTβR deficient mice have a differential susceptibility to PbA infection. However, we did not find any difference in susceptibility of male and female LTβR deficient mice to PbA infection unlike that reported for *P. chabaudi* infection [29].

Since members of the TNF family are involved in lymphoid organogenesis, we next questioned whether ECM resistance might be related to lymphoid organ structure and function. ECM resistant LTα, LTαβ or LTβR deficient mice lack lymph node (LN) formation, while partially protected LTβ deficient mice retain mesenteric LNs and in some cases cervical LNs, and intact lymphoid organs develop in the ECM sensitive LIGHT deficient mice [18]. Therefore, although absence of LTβR led to a severe defect in LN organogenesis, mice singly deficient for either LTβ or LIGHT retained mesenteric LN, suggesting a redundancy or partial compensation of the two ligands. Bone marrow reconstitution is classically used to separate the effects on lymphoid organogenesis from the functional defects in adult mice. Wild-type bone marrow reconstitution in TNF/LTα deficient mice, that corrects immunodeficiency but not LN formation [24], could not restore their sensitivity to ECM [10]. Although the potential contribution of radiation-resistant brain endothelial cells, microglial cells or astrocytes as sources of LTα was proposed, the involvement of immune defects such as LN formation cannot be ruled out. We thus investigated in more details the inflammatory and hematological responses to PbA infections.

LTβR deficient mice, similar to wild-type mice, showed thrombocytopenia and decreased lymphocyte counts at day 7 when mice show signs of incipient CM, together with a slight anemia and slight increase in parasitaemia, that further increased thereafter. Therefore, disruption of LTβR signaling while protecting from ECM development, had no strong effect on the typical hematological / inflammatory response to PbA infection in mice. Further, absence of LTα or LTβR did not interfere with the progression of blood stage infection, and LTα or LTβR deficient mice succumb within 3 weeks of severe parasitaemia and anaemia.

Recent investigations demonstrated a potential for innate immune mechanisms directed against *Plasmodium* parasites to contribute to pathology and protection from malaria [30]. Several cell types such as DC, macrophages, γδ T, NK and NKT cells contribute to the innate response [31]. Among them, non lymphoid hematopoietic cells, but also follicular dendritic cells and stroma cells express LTβR. Both human and murine DC are activated by blood stage parasites in a TLR9 dependent manner [32], which is possibly mediated by hemozoin derived from infection with the human parasites [33]. NK cell are an important source of IFNγ and disruption of IFNγ signaling or NK cell depletion leads to increased parasitaemia and higher mortality in mouse models of malaria [31], [34]. We have recently shown that macrophage derived IL-18 is required for NK cell activation by *P. falciparum* infected erythrocytes resulting in IFNγ production, which depends on IL18R/MyD88 signaling on both macrophages and NK cells [35]. However, TLR signaling of NK cells is dispensable for their activation [35]. We extensively investigated the role of TLR and their adaptor proteins, in the control of ECM, but found that TLR signaling does not contribute to the development of ECM induced by blood-stage PbA [36] while others suggested TLR9-MyD88 dependence of ECM development.

CD8^+^ T cells were recently shown to be the primary effectors of CM in the *Plasmodium berghei* ANKA mouse model, although their precise mechanism of action remains unresolved [37]. CD8^+^ T cells may mediate circulatory shock, vascular permeability changes, and edema in the brain during PbA infection [38]. We therefore investigated whether absence of LTβR affects T cell response to PbA induced ECM, and whether the effect is direct. We show a clear reduction in brain-infiltrating CD8^+^ cells in ECM resistant LTβR mice. The low recruitment and activation of CD8^+^ T cells in the brain of LTβR deficient mice may be linked to a defective production of CXCR3 ligands CXCL9, 10, 11, essential for T cell migration to inflamed tissues, that are reduced in the absence of LTβR [11]. Absence of LTβR seemed to have little incidence on the effector properties of the sequestered CD8^+^ T cell which expressed perforin, although the overall population of perforin^+^ CD8 cells was clearly reduced in LTβR deficient mice, due to a sharp decrease in CD8+ population recruited in the brain. ICAM^+^ and CD69^+^ CD8^+^ T cells were also largely reduced in the brain of PbA infected LTbR deficient mice. LTα/β expressing CD8^+^ T cells might participate directly in the microvascular pathology, through an interaction with LTβR expressed on endothelial cells, leading to cell activation, expression of adhesion molecules or cytokine secretion. The diminished recruitment and activation of CD8^+^ T cells in the brain of LTβR deficient mice may therefore be responsible for protection from ECM development.

Bone marrow irradiation chimera experiments revealed that LTβR expression on radioresistant resident stromal cells is essential for the development of ECM. Reconstitution of LTβR deficient mice with wild-type bone marrow did not confer sensitivity to ECM. Therefore, the data suggest that LTβR expressed on endothelial cells might be critical, and their absence may prevent the recruitment of CD8 lymphocytes into the brain and microvascular pathology.

The clinical relevance of the findings in mice are under investigation for gene polymorphism of the TNF/LTα locus [39]. Association between TNF/LTα polymorphism and increased risk of cerebral malaria has been reported [40]–[43], however more investigations are necessary to fully decipher the molecular bases of the link between disease manifestation and TNF/LTα polymorphism.

In conclusion, the data suggest that both functional LTβR and TNFR2 signaling are required and non-redundant for the development of microvascular pathology resulting in fatal ECM. The disruption of either axis prevents cerebral microvascular disease.

## Materials and Methods

### Mice

Mice deficient for TNF [44], LTα [45], LTβ [46] and LTαβ [47], TNFR1 [48], TNFR2 [49], LTβR [17] or LIGHT deficient mice [18] were bred in the animal facility of the Transgenose Institute (CNRS, Orleans). All mice were on C57BL/6J genetic background (back-crossed at least 10 times, beside TNFR2 deficient mice that were back-crossed 2 times) and wild-type control C57BL/6J (WT) mice were used. For experiments, adult (7–10 weeks old) mice were kept in filtered-cages in P2 animal facility. All animal experiments complied with the French Government's ethical and animal experiments regulations.

### Experimental malaria infection

A cloned line of *Plasmodium berghei* ANKA (PbA) stably transfected with GFP was obtained from Dr. A Waters [50]. Mice were infected by intraperitoneal injection of 10^6^ parasitized erythrocytes as described before [9]. Mice were observed daily for clinical neurological signs of ECM culminating in ataxia, paralysis and coma. Parasitaemia was analyzed by flow cytometry [22] at various time point and hematological parameters were assessed as indicated. Before succumbing to neurological symptoms, mice were killed for histological analysis of the brain, lung and liver.

### Parasitaemia

Parasitaemia was assessed in 2 µl of blood collected from the tail vein at different time points post-infection with EGFP-Plasmodium berghei ANKA. Blood was diluted in 2 ml of PBS containing 0.5% BSA and fluorescent cells analyzed by FACScan flow cytometer LSR (Becton Dickinson, Grenoble, France) using CellQuest software (Becton Dickinson, Grenoble, France).

### Hematology

Blood was drawn into tubes containing EDTA (Vacutainer; Becton, Grenoble, France) as indicated and hematological parameters determined using 5-part-differential hematology analyzer MS 9.5 (Melet Schloesing Laboratoires, France).

### Histology

For histological analysis, the mice were perfused under anesthesia with saline and the brain, lung, and liver from *P berghei* ANKA infected mice were fixed in 4% phosphate-buffered-saline formaldehyde for 72 h as described before [9]. Three µm sections were stained with haematoxylin and eosin. CNS microvascular obstruction, erythrocyte accumulation in alveoli, thickening of alveolar septae were quantified using a semi-quantitative score with increasing severity of changes (0–5) by two independent observers including a trained pathologist (BR) as described [9].

### Bone marrow chimera

C57BL/6J (WT) mice and LTβR deficient mice received lethal body irradiation (8 Gy). Fresh, nucleated bone marrow cells (5×10^6^ per mouse) were injected into the lateral tail vein of the recipient mice 24 hours after irradiation. The reconstituted mice were used at 2 months after bone marrow transplantation [24], [25].

### Assessment of vascular leak

Mice were injected intravenously (i.v) with 0.2 ml of 1% Evans blue (Sigma-Aldrich) on day 7, shortly before the death of the wild type mice. One hour later, mice were sacrificed and the coloration of brain was assessed. Brain and lung extravasation in formamide (Sigma-Aldrich) was evaluated by absorbance at 610 nm of the tissue extracts as an indicator of increased capillary permeability [9].

### Brain imaging using magnetic resonance imaging (MRI)

MRI experiments were performed in a horizontal 9.4 T/20-ECM Bruker Biospec MR system (Bruker Biospin, Wissembourg, France) equipped with gradients capable of switching 950 mT/m in 50 µs. A 12 elements linear birdcage coil (Bruker Biospin) with inner diameter of 35 mm and length 60 mm has been used to achieve uniform excitation and reception.

A custom-built stereotaxic head holder was used to fix the animals into the birdcage coil (see below). The mice were anaesthetized with isoflurane (1.5–2.5%) in an O2/N2O 1∶1 mixture applied with a face mask allowing free breathing. Respiration was monitored using a balloon taped to the abdomen and connected to a pressure transducer (SA Instruments, Inc., Stony Brook, NY, USA). The mice body temperature was kept at 37±0.5 °C throughout the experiment, using warm water circulation

Preparatory anatomical saggital MR images were performed using a MSME (multi-slices, multi-echoes) sequence, with the following parameters: RARE factor = 8, TR/TE_eff._ = 4000/46 ms, FOV = 20×20 mm^2^, matrix = 128×128, 1 slice 1 mm thickness, number of averages (NA) = 1, experimental time = 1 min.

Brain lesions and global changes in tissue structure have been accessed by T_2_ weighted (T2w) MR images using a MSME sequence, with the following parameters: RARE factor = 8, TR/TE_eff._ = 5000/46 ms, FOV = 17×17 mm^2^, matrix = 256×192 (zero-filled to 256×256), 37 axial slices 0.5 mm thickness, NA = 8, experimental time = 16 min.

Measurements of vascular cerebral blood flow was performed by MR angiography (MRA) using a Fast Low Angle Shot (FLASH) sequence, with the following parameters: FOV = 14×14 mm^2^, matrix = 128×128, TR/TE = 30/5 ms, 51 axial slices 0.3 mm thickness, NA = 4, experimental time = 13 min. Angiograms were produced by generating maximum intensity projections (MIP) after interpolating raw data to obtain an isotropic resolution (109 µm)^3^. Image analysis and processing were performed with the public domain software Image J (NIH, http://rsb.info.nih.gov/ij).

### Isolation of brain- sequestered leukocytes

Mice with neurological symptoms of ECM were anaesthetized (at day 7–8) and perfused with 10 ml of PBS/EDTA 0.02% intra-cardiac to remove circulating red blood cells and leukocytes from the brain as described.[20] Brains were removed and homogenized gently (30 sec at 4000 rpm) using the sterile disposable homogenization system Dispomix (Medic Tools AG, 6300-Zug, Switzerland) in RPMI 1640 medium containing 2% FCS. Tissue-cell homogenates were passed through a nylon cell strainer (100 µm; Becton Dickinson Grenoble, France) and centrifuged at 400 g for 10 minutes. The mononuclear cells were then separated over a 30% Percoll gradient (Amersham Biosciences AB, Uppsala, Sweden). Residual red blood cells were lyzed by hypotonic shock in ammonium chloride- potassium lysis buffer.

### Flow cytometry analysis (FACS)

Brain-sequestered cells were collected, washed and saturated with mouse serum prior to the staining with fluorescence labelled antibodies for 30 minutes. The cells were then phenotyped by flow cytometry. T cells were identified by their forward side scatter and with the use of the following antibodies: rat anti-mouse CD4 conjugated to fluorescein isothiocyante (FITC) (clone GK1.5; Pharmingen San Diego, CA), rat anti-mouse CD8α conjugated to allophycocyanin (APC) (clone 53-6.7, Pharmingen San Diego, CA), rat anti-mouse Perforin conjugated to phycoerytthrin (PE) (clone JAW246, eBioscience San Diego, CA), rat anti-mouse CD69 conjugated to phycoerytthrin (PE) (clone H1,F3, Becton-Dickinson, Grenoble France), rat anti-mouse CD54 conjugated to PE (clone 3E2, Becton-Dickinson, Grenoble France) and isotype-matched control antibodies (Becton-Dickinson, Grenoble France). For each sample, 10000 cells were scored. Data were analyzed using a LSR II-Flow cytometer and CellQuest software (Becton-Dickinson, Grenoble France).

### Statistical analysis

Data are presented as mean values and standard deviation (SD) indicated by error bars, if not otherwise indicated. Statistical significance was determined with Graph Pad Prism software (version 4.0, San Diego,CA). Differences between multiple groups were analyzed for statistical significance by means of one way non-parametric ANOVA test followed by Dunn's multiple comparison test for some data or Logrank Test, for others we used Kruskal-Wallis test followed by Dunn's multiple comparison test. P values of <0.05 were considered statistically significant.

## Supporting Information

Video S1 3D MRA Video 1 : Non-infected C57BL/6 wild-type mouse. Three-dimensional (3D) Magnetic Resonance Angiography (MRA) movies of non-infected wild-type C57BL/6, and wild-type or mice deficient for TNF, LTαLTβ, or LTβR 7 days post infection with 10e6 P. berghei ANKA parasitized red blood cells. Video 1 : Non-infected C57BL/6 wild-type mouse. Compared to 2D views, 3D MRA allows a better observation of the vascular blood flow perturbations in wild-type and TNF-KO mice. Before 3D reconstruction, two dimensional angiograms (2D-MRA) were produced by Maximum Intensity Projections (MIP) as described in the Methods section. Movies were created by rotating the 2D-MRA views at different angles (using steps of 20 degrees). Image analysis and processing (including 3D MRA movies) were performed with the public domain software ImageJ (NIH, http://rsb.info.nih.gov/ij).
(1.71 MB AVI)

Video S2 3D MRA Video 2 : PbA-infected C57BL/6 mouse. Three-dimensional (3D) Magnetic Resonance Angiography (MRA) movies of non-infected wild-type C57BL/6, and wild-type or mice deficient for TNF, LTα ~LTβ, or LTβR 7 days post infection with 10e6 P. berghei ANKA parasitized red blood cells. Video 2 : PbA-infected C57BL/6 mouse. Compared to 2D views, 3D MRA allows a better observation of the vascular blood flow perturbations in wild-type and TNF-KO mice. Before 3D reconstruction, two dimensional angiograms (2D-MRA) were produced by Maximum Intensity Projections (MIP) as described in the Methods section. Movies were created by rotating the 2D-MRA views at different angles (using steps of 20 degrees). Image analysis and processing (including 3D MRA movies) were performed with the public domain software ImageJ (NIH, http://rsb.info.nih.gov/ij).
(1.92 MB AVI)

Video S3 3D MRA Video 3 : PbA-infected TNF ko mouse. Three-dimensional (3D) Magnetic Resonance Angiography (MRA) movies of non-infected wild-type C57BL/6, and wild-type or mice deficient for TNF, LTα ~LTβ, or LTβR 7 days post infection with 10e6 P. berghei ANKA parasitized red blood cells. Video 3 : PbA-infected TNF ko mouse. Compared to 2D views, 3D MRA allows a better observation of the vascular blood flow perturbations in wild-type and TNF-KO mice. Before 3D reconstruction, two dimensional angiograms (2D-MRA) were produced by Maximum Intensity Projections (MIP) as described in the Methods section. Movies were created by rotating the 2D-MRA views at different angles (using steps of 20 degrees). Image analysis and processing (including 3D MRA movies) were performed with the public domain software ImageJ (NIH, http://rsb.info.nih.gov/ij).
(1.92 MB AVI)

Video S4 3D MRA Video 4 : PbA-infected LTαβ ko mouse. Three-dimensional (3D) Magnetic Resonance Angiography (MRA) movies of non-infected wild-type C57BL/6, and wild-type or mice deficient for TNF, LTαLTβ, or LTβR 7 days post infection with 10e6 P. berghei ANKA parasitized red blood cells. Video 4 : PbA-infected LTαβ ko mouse. Compared to 2D views, 3D MRA allows a better observation of the vascular blood flow perturbations in wild-type and TNF-KO mice. Before 3D reconstruction, two dimensional angiograms (2D-MRA) were produced by Maximum Intensity Projections (MIP) as described in the Methods section. Movies were created by rotating the 2D-MRA views at different angles (using steps of 20 degrees). Image analysis and processing (including 3D MRA movies) were performed with the public domain software ImageJ (NIH, http://rsb.info.nih.gov/ij).
(1.74 MB AVI)

Video S5 3D MRA Video 5 : PbA-infected LTβR ko mouse. Three-dimensional (3D) Magnetic Resonance Angiography (MRA) movies of non-infected wild-type C57BL/6, and wild-type or mice deficient for TNF, LTαLTβ, or LTβR 7 days post infection with 10e6 P. berghei ANKA parasitized red blood cells. Video 4 : PbA-infected LTβR ko mouse. Compared to 2D views, 3D MRA allows a better observation of the vascular blood flow perturbations in wild-type and TNF-KO mice. Before 3D reconstruction, two dimensional angiograms (2D-MRA) were produced by Maximum Intensity Projections (MIP) as described in the Methods section. Movies were created by rotating the 2D-MRA views at different angles (using steps of 20 degrees). Image analysis and processing (including 3D MRA movies) were performed with the public domain software ImageJ (NIH, http://rsb.info.nih.gov/ij).
(1.71 MB AVI)
